# Alterations of gut microbiome following gastrointestinal surgical procedures and their potential complications

**DOI:** 10.3389/fcimb.2023.1191126

**Published:** 2023-06-02

**Authors:** Christina Tsigalou, Afroditi Paraschaki, Nicola Luigi Bragazzi, K. Aftzoglou, Elisavet Stavropoulou, Z. Tsakris, S. Vradelis, Eugenia Bezirtzoglou

**Affiliations:** ^1^ Laboratory of Microbiology, Faculty of Medicine, Democritus University of Thrace, Dragana Campus, Alexandroupolis, Greece; ^2^ Department of Biopathology/Microbiology, Faculty of Medicine, University General Hospital of Alexandroupolis, Alexandroupolis, Greece; ^3^ Laboratory for Industrial and Applied Mathematics (LIAM), Department of Mathematics and Statistics, York University, Toronto, ON, Canada; ^4^ Medical School, Comenius University, Bratislava, Slovakia; ^5^ Department of Infectious Diseases, Centre Hospitalier Universitaire Vaudois (CHUV), Rue du Bugnon, Lausanne, Switzerland; ^6^ Laboratory of Microbiology, Department of Medicine, National and Kapodistrian University of Athens, Athens, Greece; ^7^ Department of Gastrenterology, Faculty of Medicine, Democritus University of Thrace, Dragana Campus, Alexandroupolis, Greece; ^8^ Laboratory of Hygiene and Environmental Protection, Medical School, Democritus University of Thrace, Dragana, Alexandroupolis, Greece

**Keywords:** gut microbiota, microbiome, surgery complications, surgical disease, alterations in microbiota, peri-operative interventions

## Abstract

Intestinal microorganisms play a crucial role in shaping the host immunity and maintaining homeostasis. Nevertheless, alterations in gut bacterial composition may occur and these alterations have been linked with the pathogenesis of several diseases. In surgical practice, studies revealed that the microbiome of patients undergoing surgery changes and several post-operative complications seem to be associated with the gut microbiota composition. In this review, we aim to provide an overview of gut microbiota (GM) in surgical disease. We refer to several studies which describe alterations of GM in patients undergoing different types of surgery, we focus on the impacts of peri-operative interventions on GM and the role of GM in development of post-operative complications, such as anastomotic leak. The review aims to enhance comprehension regarding the correlation between GM and surgical procedures based in the current knowledge. However, preoperative and postoperative synthesis of GM needs to be further examined in future studies, so that GM-targeted measures could be assessed and the different surgery complications could be reduced.

## 1 Introduction

Microbiome is a complex and dynamic ecosystem composed of a dense microbial population, in which hundreds of microbial species coexist (Wang et al., 2019). The average human body harbors 100 trillion of microorganisms, both inside and out, with the vast majority located in gastrointestinal (GI) tract (NIH HMP Working Group et al., 2009; Ursell et al., 2012). In fact, GI tract harbors 10 times more bacterial cells than human cells and carries 150 times more genes (microbiome) than the entire human genome (Thursby and Juge, 2017; Tsigalou et al., 2021). Over centuries, humans have ignored the importance of those microbial organisms which are proven to be essential for their wellbeing. Research has shown that humans live in close relationship with boundless communities of microorganisms that live on and within human bodies and play a major role in human health and disease (Tsigalou et al., 2021). In relation to that, in 2007 the Human Microbiome Project, consisting of multiple projects worldwide, started a research project using sequencing methods to characterize and describe the human microbiome and analyze how it can affect human health and disease (Turnbaugh et al., 2007; Tsigalou et al., 2021). Subsequently, there are several recent technologies of genomics sequencing, proteomics and metabolomics for the identification and analysis of GM (Alverdy et al., 2017).

In recent years, there has been a growing interest in the field of human gut microbiota (GM). Under normal conditions, GM prevents pathogens from crossing the intestinal barrier. Besides, it has been shown to contribute to prevention of nosocomial infections. On the other hand, a disruption to the microbiota homeostasis seems to be involved both in disease onset and development of complications after surgical procedures. Various studies have shown that there is an association between GM and certain diseases, such as inflammatory bowel disease (IBD) including Crohn’s disease (CD) (Shimizu et al., 2011; Zaborin et al., 2014; Stavrou and Kotzampassi, 2017; Schmitt et al., 2019). Surgery turns out to have a significant impact on the GM with a great number of medical and surgical problems to be linked to perturbations of the microbiome (Morowitz et al., 2011). For instance, Schmitt et al. (Schmitt et al., 2019) shows that pancreatic surgery affects the GM, while Fang et al. (Fang et al., 2021) describes another types of surgery which affect th GM as well. Therefore, despite the improvement in operation techniques and the quality of general surgical care, postoperative complications remain a notable problem and a considerable number of patients experience postoperative morbidity. Patient age, medical comorbidities, longer procedural times, even the type of surgery are some of the well-recognized risk factors. Nevertheless, postoperative complications may occur even if a low number of risk factors exists. In recent years, the use of Next-generation sequencing (NGS) helped researchers to identify the intestinal microbial composition. NGS allows high-throughput sequencing of DNA samples, so that large numbers of bacterial genomes can be sequenced rapidly in a single experiment. Hence, by using these NGS techniques, researchers can better understand the different bacterial populations and see how microbial imbalances can lead to various health diseases. (Slatko et al., 2018; Gupta and Verma, 2019; Galloway-Peña and Hanson, 2020). Consequently, GM has been shown to play a crucial role in occurrence of postoperative complications (Schmitt et al., 2019). Thus, ongoing research has the potential to lead to new strategies which may enhance the outcomes of surgical procedures.

The key words surgery, microbiota and microbiome’ were used to search for relevant studies published in Pubmed database between 2011 and 2022. After evaluation of the full text of the articles, the articles were selected according to the main topics of this review, which are gut microbial dysbiosis linked with human diseases, alterations of GM following surgery, impact of peri-operative interventions on GM and post-operative complications related to GM.

## 2 Gut microbial dysbiosis and surgical disease

The human gastrointestinal tract harbors several species of microorganisms, including bacteria, fungi, and viruses. The main microbial phyla present in GI tract are Firmicutes (e.g. *Clostridium, Lactobacillus*) and Bacteroidetes (e.g. *Bacteroides, Prevotella*) representing around 60% of gut microbiota, followed by Actinobacteria, Proteobacteria and Fusobacteria (Arumugam et al., 2011; Thursby and Juge, 2017; Rinninella et al., 2019). Each individual can be described with a unique GM profile while a balanced gut microbiota composition confers benefits to the host. The microbiota collaborates with the host’s defenses and immune system to protect against pathogen invasion. Furthermore, it exerts profound influence on host metabolism by taking part in digestion of food ingredients leading to essential nutrients and vitamins production (Vernocchi et al., 2020). Contrariwise, imbalance of gut’s microbial community, a condition called dysbiosis, alters the physiological functions of the host and, as a result, is associated with unhealthy outcomes and leads to pathogenesis of common human diseases. Dysbiosis contributes to the development of various disorders, including IBD, metabolic syndrome, diabetes, and cancer among many others (Carding et al., 2015; Belizário and Faintuch, 2018; Martinez et al., 2021). Hence, a broad range of surgical problems have been linked to disturbance of the GM composition.

### 2.1 IBD

IBD, which includes CD and ulcerative colitis (UC), is a chronic, progressive immune-mediated disease affecting the gastrointestinal tract and has become a global emergence disease(M’Koma, 2013). It is estimated that 0.3% of the European population has been diagnosed with IBD, which means that, approximately, a total of 2.5-3 million people is affected. In North America its prevalence is estimated to already exceed 0.5% of the population (Burisch et al., 2013; Coward et al., 2019; Hammer and Langholz, 2020).

Several factors, environmental and immunologic, can lead genetically susceptible hosts to inflammation. More recently, studies have associated alterations in GM with occurrence of IBD. Advances in cultivation-independent technologies showed decreased biodiversity of the gut microflora in those patients and intestinal dysbiosis has been well described (Ott et al., 2004; Manichanh et al., 2006; Frank et al., 2007; Dalal and Chang, 2014). In IBD, Enterobacteriaceae are enriched in the microbial flora and adherent-invasive *Escherichia coli* is commonly isolated from biopsy samples of those patients with CD (Darfeuille-Michaud et al., 2004). Neut et al (Neut et al., 2002) demonstrated that patients undergoing ileocecectomy are more likely to develop postoperative recurrence of CD when high counts of *E.coli* and *Bacteroides* were present. Thus, microbiome seems to play a significant role in development and progression of IBD (Manichanh et al., 2006; Dalal and Chang, 2014; Skowron et al., 2018; Glassner et al., 2020).

Several mutations in genes related to immune system are involved in microbiome-immune interactions and, therefore, in pathogenesis of IBD. It is clearly shown that there is a connection between intestinal flora and intestinal immune cells. Nucleotide oligomerization domain 2 (NOD2), for instance, plays an important role in immune function by recognizing bacterial cell wall proteins and contributing to commensal microbes’ control in gut. Mutations in NOD2 gene is a strong genetic risk factor in the pathogenesis of IBD. NOD2- deficient mice have an altered microbiome and increased susceptibility to colitis, sensitizing the colonic mucosa to injury (Kobayashi et al., 2005; Petnicki-Ocwieja et al., 2009; Couturier-Maillard et al., 2013; Skowron et al., 2018).

Moreover, other risk factors related to the microbiome predispose the host to the development of IBD. Use of antibiotics can alter the composition of GM and a study in Denmark (Hviid et al., 2011) showed that early exposure to antibiotics in childhood can lead to IBD and CD. Dietary habits also influence the intestinal flora and play a significant role in shaping its composition. High-fat diets lead to dysbiosis, while plant-based diets affect the GM positively (Tsigalou et al., 2021). Hou et al (Hou et al., 2011) found that diets based in high intake of fats and protein are associated with increased risk of IBD, while high fiber, fruit and vegetable consumption were associated with decreased CD and UC risk. Diet-induced shifts in GM can explain those findings. In addition, breast-feeding seems to be protective against development of IBD. The microbial diversity in breast milk promotes immune tolerance and prevents infections (Xu et al., 2017). In general, a microbial-centered etiology is proposed to explain the development of IBD.

### 2.2 Colorectal cancer

Dysbiosis of gut microbiota is closely related to colorectal cancer (CRC). CRC is one of the most common types of cancer worldwide. It ranks third in terms of incidence and is the second most common cause of cancer death. Nearly 2 million new cases were diagnosed in 2020 and almost 1 million deaths occur per year (*Colorectal Cancer Awareness Month 2022 – IARC*, no date).

Recent reports have demonstrated that GM plays a crucial role in progression of CRC. Studies have shown alterations in the intestinal microbiota synthesis of patients with reduced bacterial diversity compared with healthy individuals (Chen et al., 2012). Also, several bacterial species have been associated with CRC. *Streptococcus bovis*, enterotoxigenic *Bacteroides fragilis*, *Fusobacterium nucleatum*, *Enterococcus faecalis* and biofilms with species of *E.coli* are some of them (Chen et al., 2012; Wang et al., 2012; Kostic et al., 2013; Sears et al., 2014; Denizot et al., 2015; Veziant et al., 2016; Cheng et al., 2020).

Chronic inflammation is accepted as a risk factor for CRC and, therefore, patients with IBD are in higher risk of CRC development. Intestinal microbiota interacts, as mentioned, with the host immune system and, subsequently, immune responses to bacteria can lead to low-grade inflammation which can lead to tumorigenesis (Arthur et al., 2012). Furthermore, damaged host protective barriers, like intestinal epithelium in colitis, allow translocation of bacteria and exposure to bacterial products. The host though may respond by producing pro-inflammatory cytokines, such as IL-17, -23, TNF-a, which have the characteristic to be also pro-tumorigenic (Garrett, 2015; Skowron et al., 2018). Dejea et al. (Dejea et al., 2014) suggested that colon mucosal biofilm formation enhanced bacterial translocation across the gut barrier due to greater epithelial permeability, which promotes inflammation and may predict increased risk for CRC.

### 2.3 Obesity

Obesity is a complex metabolic disorder and a result of both genetic and environmental factors. Moreover, studies revealed that obesity is closely related to GM (Liu et al., 2021). It has been shown that GM differs in obese individuals. The microbial composition seems to show low diversity and variety in obese people, with an overgrowth of Gram-negative pathogens which promote lipopolysaccharides (LPS) diffusion and causing low grade chronic inflammation and increased intestinal permeability leading to obesity (Vallianou et al., 2019; Tsigalou et al., 2021; Zsálig et al., 2023). Many studies of the gut microbiome of obese individuals revealed significant alterations of intestinal bacterial phyla with increase in Firmicutes and decrease in the abundance of Bacteroidetes leading to an elevated Firmicutes/Bacteroidetes ratio (Ley et al., 2005). Furthermore, reduced abundance of *Bifidobacterium* is associated with obesity, whilst Streptococcaceae are associated with those individuals with higher BMI (Waldram et al., 2009) (Garcia-Mantrana et al., 2018). Other studies summarized the effect of *Lactobacillus* on body weight and found that *Lactobacillus paracasei* was reduced in overweight subjects, while *Lactobacillus reuteri* and *Lactobacillus gasseri* were increased(Million et al., 2012; Crovesy et al., 2017). Million et al. (Million et al., 2012) showed, also, reduced levels of *Methanobacteriales smithii* in obesity.

Therefore, dysbiosis of GM has been shown to be linked to obesity. However, further research in that field is needed to understand the interaction between GM and obesity.

### 2.4 Type 2 diabetes

Type 2 diabetes (T2D) is a metabolic disorder which has become a global health problem. While there are various risk factors associated with the development of T2D, emerging evidence suggests that gut microbiota may play a significant role in the development of this disease. Disturbances of GM may increase gut permeability and lead to signaling pathways, related to the insulin resistance in T2D patients. Therefore, research has shown a reduction in beneficial bacteria such as *Bifidobacteria* and an increase in Firmicutes (Sharma and Tripathi, 2019; Zhou et al., 2022)

Furthermore, GM can produce various metabolites that can impact glucose and lipid metabolism. For instance, short-chain fatty acids (SCFAs) produced by gut bacteria can affect insulin signaling and glucose metabolism. Nevertheless, dysbiosis has been associated with altered SCFA production in individuals with type 2 diabetes(Portincasa et al., 2022; Zhou et al., 2022).

Overall, further research is needed to fully understand the relationship between gut microbiota and type 2 diabetes so that potential therapeutic interventions that target the gut microbiota may improve glucose metabolism and insulin sensitivity.

## 3 Alterations in the Gastrointestinal Microbiome after surgery

Surgery has a large effect in the microbiome and can profoundly alter the synthesis of gut microbiota (Morowitz et al., 2011). Great number of studies have shown that surgical operations are associated with changes in microbiota composition.

Among studies that looked at IBD, Fang et al. (Fang et al., 2021) reported that intestinal surgeries reduce the diversity of GM in IBD patients. A total of 332 stool samples from 129 subjects suffering from UC or CD were collected. Both species and metabolite diversity differed among groups with a significant decrease in phylogenetic diversity after ileocolonic resection and colectomy. In addition, there was a significant increase in the expansion of *E.coli* in individuals who underwent surgery and, in particular, samples from IBD patients who underwent colectomy had the highest abundance of *E.coli*. Wright et al. (Wright et al., 2017) also indicated that there is a significant difference in bacterial composition 6 months and 18 months after surgery. A total of 141 mucosal biopsy samples from 34 CD patients were collected at surgical resection and at colonoscopy after surgery. In addition, 28 control samples were obtained. At 6 months, endoscopic recurrence was associated with elevated *Proteus* genera and at 18 months with reduced *Faecalibacterium*, *Desulfovibrio* and *Bilophila* abundance. At 18 months severe endoscopic recurrence was associated with increase in Proteobacteria. In patients with subsequent remission significant increases in the Firmicutes phylum, the Bacteroidaceae and Pasteurellaceae families and *Bacteroides* genus were observed when compared to those with recurrence. Mondot et al. (Mondot et al., 2016) showed that ileocolonic resection (ICR) in CD patients had a dramatic impact on gut microbial ecosystem. Twenty patients were included in the study. Six months after surgery, ten patients developed recurrence of CD lesions. Samples collected at time of surgery were enriched in Proteobacteria and harbored high levels of unusual, bacteria such as *Streptococcus mitis*, *Undibacterium oligocarboniphilum*, *Sphingomonas melonis* and *Gemella haemolysans*, while 6 months after surgery samples had elevated proportions of anaerobic bacteria belonging to Lachnospiraceae (*Clostridium nexile*, *Blautia wexlereae*, *Dorea longicatena*). Remission was characterized by increased levels of bacteria belonging to *Bacteroides*, *Dorea*, *Ruminococcus* and *Dialister* genera, whereas recurrence was associated with increased levels of *Gemmiger formicilis*, *Enterococcus durans* and *Ruminococcus lactaris*.

Furthermore, other studies demonstrate changes in the balance within the intestinal microbiota after colorectal surgery. Deng et al. (Deng et al., 2018) reported reduction of Bacteroidetes and Firmicutes and increase of Proteobacteria in patients undergoing surgery for CRC. Sze et al. (Sze et al., 2017) showed differences to the bacterial community after surgery in patients treated for adenomas or carcinomas. Microbial communities from patients with carcinomas changed more notable than those with adenomas after surgical treatment and were more similar to those of healthy people after surgery. Komatsu et al. (Komatsu et al., 2016) reported a significant reduction in the total number of bacteria and the number of dominant obligate anaerobes (such as *Clostridium coccoides* group, *Clostridium leptum* subgroup, *B. fragilis* group, *Bifidobacterium*, *Prevotella*, and *Lactobacillus* species) and increase in the abundance of Enterobacteriaceae, *Staphylococcus* (Methicillin-Susceptible Coagulase-Negative Staphylococci MSCNS), *Pseudomonas*, and *Clostridioides difficile* after colorectal surgery. Feng et al. (Feng et al., 2015) collected both faecal and mucosal samples before and six months after colorectal surgery. The abundance of bifidobacterial and lactobacilli in feces and the number of bifidobacterial in mucosa increased after surgery, while Firmicutes and Bacterioidetes decreased after surgery.

Schmitt et al. (Schmitt et al., 2019) analyzed 116 stool samples from 32 patients undergoing pancreatic surgery and examined changes in GM following pancreatic surgery. The samples were classified into three different microbial communities (A, B, C). Community B showed increase in *Akkermansia*, *Aeromonas*, Enterobacteriaceae and Bacteroidales and decrease in Lachnospiraceae, *Prevotella* and *Bacteroides*. Schmitt et al. also observed that the majority of patients experiencing complications showed microbial community B during the period after surgery.

Other studies assessed the remodeling of gut microbiota after bariatric surgery. Steinert et al. (Steinert et al., 2020) found that Roux-en-Y gastric bypass (RYGB) resulted in clear alteration in composition of gut fungal and bacterial microbiota. Before surgery, patients had higher levels of Firmicutes and Actinobacteria and lower levels of Verrucomicrobia in comparison with healthy individuals, which changed after surgery as the levels of those bacterial groups were not different anymore. Also, *Faecalibacterium* and *Bifidobacterium* showed a decrease after surgery and aerotolerant *Streptococcus* was increased postoperatively. The relative abundance of Proteobacteria was increased after surgery. Changes in fungal microbiota composition included decreases in *Candida* and *Saccharomyces* and increases in *Pichia*. Assal et al. (Al Assal et al., 2020) reported that gut microbial richness increased after RYGB and the Firmicutes to Bacteroidetes ratio decreased. Shen et al. (Shen et al., 2019) showed that the levels of Verrucomicrobia and Proteobacteria increased after the surgery procedure and despite previous studies no changes were noticed in Firmicutes and Bacteroidetes. Paganelli et al. (Paganelli et al., 2019) determined alterations in microbiota composition in 45 obese patients who underwent crash diet followed by RYGB or sleeve gastrectomy (SG). Bifidobacteriaceae abundance decreased, whereas Streptococcaceae and Enterobacteriaceae increased after surgery and no significant differences appeared between both types of surgery in contrast to other studies (Liou et al., 2013). Murphy et al. (Murphy et al., 2017) examined gut microbiota changes after RYGB and SG surgery in obese patients with type 2 diabetes. RYGB led to increased Firmicutes and Actinobacteria phyla but decreased Bacteroidetes phylum. SG resulted in significantly increased Bacteroidetes phylum.

Overall, the results of those studies indicate that there is accumulating evidence that the microbiota can be significantly altered in patients undergoing different types of surgery. Changes following surgery procedures are noticed both in diversity and in the numbers of specific bacteria. **Table 1** contains a summary of the articles mentioned in this review.

**Table 1:** Representative studies linking the type of surgery with alterations of GM.

Study Design	Type of surgery	Year	Alteration of Microbial Population	Outcome	References
Prospective longitudinal study	Surgery for IBD: Ileocolonic resection and colectomy	2020	Surgery lowered diversity and affected overall taxonomic, functional and metabolite profiles Elevated *E. coli* relative abundance in surgery samples	Long-term effect of intestinal surgeries on the gut microbiome of IBD patients	Fang, X. et al. (Fang et al., 2021)
Prospective, randomised, controlled trial	Surgery for IBD: resection in CD patients	2017	Microbial composition changed within CD patients. Resection samples were different to samples taken at colonoscopy at 6 and 18 months. Recurrence associated with elevated *Proteus* genera and reduced *Faecalibacterium*	Microbial factors and smoking independently influence postoperative CD recurrence. The genus Proteus may play a role in the development of CD	Wright, E. K. et al. (Wright et al., 2017)
Double-blind, randomised, placebo-controlled, 6-month clinical study	Surgery for IBD: ileocolonic resection in CD patients	2016	Bacterial profiles 6 months after ICR differed markedly from the profiles at time of surgery. Remission associated with increased *Bacteroides*, *Dorea*, *Ruminococcus* and *Dialister* genera Recurrence associated with increased facultative anaerobes like *Gemmiger formicilis*, *Enterococcus durans* and *Ruminococcus lactaris*.	ICR modifies th GM.	Mondot, S. et al. (Mondot et al., 2016)
Metagenomic study of the microbiota of CRC patients after surgery and chemotherapy	Surgery for colorectal cancer	2018	Reduction of Bacteroidetes and Firmicutes. Increase of Proteobacteria	Fecal microbiome-based approaches may provide additional methods for anti-cancer treatments	Deng, X. et al. (Deng et al., 2018)
67-person cohort study	Surgery for colorectal cancer	2017	Microbial communities from carcinoma group changed more notable	Biomarkers within the microbiota could be used to potentially evaluate the effect of treatment and to predict recurrence	Sze, M. A. et al. (Sze et al., 2017)
Single-center randomized, controlled trial	Laparoscopic colorectal surgery	2016	Reduction in dominant obligate anaerobes Increase in Enterobacteriaceae, *Staphylococcus* (MSCNS), *Pseudomonas*, and *Clostridioides difficile*	The microbial imbalance, in addition to the reduction in organic acids, could be improved by perioperative synbiotics treatment	Komatsu, S. et al. (Komatsu et al., 2016)
Feces and mucosal samples of five healthy volunteers and 17 patients with refractory constipation before and six months after subtotal colectomy were collected.	Subtotal colectomy	2015	Faecal samples: increase in *Bifidobacterium* spp and *Lactobacillus* spp/decrease in Firmicutes and Bacteroides Mucosal samples: increase in *Bifidobacterium*	Subtotal colectomy has been shown to normalize the number of intestinal flora and improving refractory constipation	Feng X. et al. (Feng et al., 2015)
Prospective, observational, clinical study	Pancreatic surgery	2019	Patients showing increase in *Akkermansia*, Enterobacteriaceae and *Bacteroidales* as well as decrease in Lachnospiraceae, *Prevotella* and *Bacteroides*, at least once during the observation period were found to have a higher risk for developing postoperative complications	Sequencing of the GM might represent a useful diagnostic tool in future clinical practice	Schmitt, F. et al. (Schmitt et al., 2019)
Pilot cohort study	RYGB	2020	Changes of gut bacterial and fungal microbiota Decrease in Firmicutes, Actinobacteria, *Candida* and *Saccharomyces* Increase in Proteobacteria and *Pichia*	Changes in intestinal fungal communities in RYGB patients that are distinct to changes in the bacterial microbiota	Steinert, R. E. et al. (Steinert et al., 2020)
Prospective cohort study	RYGB	2020	GM richness increased. Firmicutes/Bacteroidetes ratio decreased	Evaluating GM profile to predict T2D remission after RYGB and modulating by dietary interventions	Assal, K. et al. (Al Assal et al., 2020)
Non-Randomized interventional study	RYGB and SG surgery	2019	Verrucomicrobia and Proteobacteria increased after surgery. No changes in Firmicutes and Bacteroidetes	Microbiome partially mediates improvement of metabolism during the first year after bariatric surgery	Shen, N. et al. (Shen et al., 2019)
Observational study	RYGB and SG surgery	2019	Decreased Bifidobacteriaceae. Increased Streptococcaceae and Enterobacteriaceae No significant differences between the two types of surgery	Crash diet invoked temporary changes in GM, yet surgery was associated with changes in GM, that likely contribute to weight loss, independent of surgery type	Paganelli, F. L. et al. (Paganelli et al., 2019)
Longitudinal study	RYGB and SG surgery	2017	RYGB: Increased Firmicutes and Actinobacteria phyla/decreased Bacteroidetes phyla SG: Increased Bacteroidetes phylum	RYGB showed greater and more predicted favourable changes in GM than SG	Murphy, R. et al. (Murphy et al., 2017)

## 4 Impact of peri-operative interventions on microbiome

As more and more studies report changes in gut microbiota in patients undergoing surgery, it turns out that many routine techniques of surgical care can affect the host microbiome and, in consequence, the clinical outcomes. There is a variety of perioperative interventions such as mechanical cleansing of the bowel, use of antibiotics and other medication or type of nutrition, along with surgical injury itself and stress of surgery, that could impact the state of the microbiome (Stavrou and Kotzampassi, 2017).

Mechanical bowel preparation (MBP) is often used before abdominal surgeries and may lead to substantial change in GM as many studies show. It seems that MBP can alter the normal flora and, hence, it can provide the opportunity to several pathogens to thrive (Morowitz et al., 2011). It may take 14 days for the intestinal microbiota composition to recover to baseline (Nagata et al., 2019). Yang et al. (Yang et al., 2022) performed a study in which a total of 81 patients were enrolled and were divided into two groups, preparation and non-preparation group, whether they received MBP before the surgery or not. The findings concluded that, at phylum level, Bacteroidetes and Fusobacteria and, at family level, Pasteurellaceae and Neisseriaceae were obviously higher in the preparation group, whereas the abundance of Lactobacillaceae and Ruminococcaceae were higher in non-preparation group. Additionally, at genus level, *Bacteroides*, *Enterobacter*, *Fusobacterium*, *Veillonella*, *Haemophilus*, and *Neisseria* among others were obviously higher in the preparation group, while *Anaerotruncus*, *Coprobacillus*, *Lactobacillus*, and *Blautia* were higher in the group of patients who did not receive MBP. Another study also detected changes in the intestinal microbiota following bowel cleansing (Jalanka et al., 2015) and revealed that, immediately after the lavage, the intestinal microbiota was significantly different when compared with the baseline samples. Bacilli and *Clostridium* cluster IV genera decreased, while members of the Proteobacteria phylum and *Clostridium* cluster XIVa showed an increased abundance. Also, a twofold increase of Proteobacteria, including *Sutterella wadsworthia* and *Serratia*, was noticed after lavage. Similarly, Drago et al. (Drago et al., 2016) found a significant decrease in Firmicutes abundance immediately after colon cleansing and an increase in Proteobacteria. Reduction in Lactobacillaceae and increase in the levels of Enterobacteriaceae were observed as well, while Streptococcaceae showed a 4-fold increase after bowel lavage.

Furthermore, the use of perioperative antibiotics – both oral and intravenous administration- can impact the microbiota leading to a significant reduction of several bacterial counts after surgery. An increase of potentially pathogenic microorganisms is reported as well (Ohigashi et al., 2013; Lederer et al., 2017; Lederer et al., 2021). Also the duration of antimicrobial prophylaxis is associated with higher rates of surgical site infections, resulting for example in higher rates of *C. difficile* infections (Branch-Elliman et al., 2019). Hedge et al. (Hegde et al., 2018) studied on rats and found that broad spectrum antibiotics dramatically reduced the total microbial abundance and diversity. The relative abundance of Firmicutes was noticed to be decreased, whereas Bacteroidetes and Proteobacteria were enriched. Similar to other studies, Nalluri et al. (Nalluri et al., 2020) tried to evaluate the impact of perioperative antibiotic administration on gut microbiome in patients undergoing vertical sleeve gastrectomy and showed that routine antibiotics led to postsurgical changes in the intestinal microbiota. Other peri-operative medications, such as antacids and opioids, can also alter the microbiome composition. Antacids are a class of medicines that neutralize stomach acidity and patients treated with high doses of omeprazole tend to decrease the microbial diversity of colon (Kostrzewska et al., 2017). A significant lower abundance in gut commensals and decreased microbial diversity is also noticed in patients treated with proton pump inhibitors (PPIs). Furthermore, PPI use was associated with increases in Streptococcaceae (Jackson et al., 2016).

Moreover, opioids have been shown to disrupt gut homeostasis. Morphine is one of the most used opioid analgesic for severe pain. In a recent study with a morphine-murine model, the results revealed a significant shift in GM after morphine treatment with the pathogenic bacteria to be increased, even in short term. A significant reduction of beneficial microorganisms, such as *Lactobacillus* and *Bifidobacterium*, was also observed (Wang et al., 2018). Gicquelais et al. (Gicquelais et al., 2020) examined gut microbiota changes related to use of opioids among people and observed that individuals exposed to opioid agonists had alterations in GM with decreased diversity and richness. Therefore, use of morphine results in alterations in gut microbiome contributing to microbial dysbiosis (Herlihy and Roy, 2022).

Increasing evidence suggest that parenteral nutrition (PN) is associated with changes in the intestinal microbiota as well. The host diet affects nutrients availability and, in consequence, the gut microbiome. Parenteral type of nutrition though is required for patients when enteral feeding is not possible (David et al., 2014; Demehri et al., 2015). In a mouse model, PN leads to a relative loss of Firmicutes and to an expansion of Proteobacteria and Bacteroidetes (Miyasaka et al., 2013). In a neonatal pig model, a significant shift in GM was also observed and was characterized by reduction in bacterial concentration throughout the intestine and loss of microbial diversity. In addition, PN-dependent piglets were at higher risk of colonization by toxin-expressing *C. difficile* (Harvey et al., 2006). When complete intravenous nutrition is applied, a nutrient-deprived environment is created for bacteria in the gut and this hostile environment may favor Proteobacteria, which have been shown to survive in relative starvation states, in contrast to Firmicutes, as they dominate in nutrient-rich environment (Demehri et al., 2015).

Some studies suggest that anesthesia can also provoke unfavorable alterations in the composition and diversity of the gut microbiota. Lian et al (Lian et al., 2021) investigated the effects of surgery and anesthesia on the gut microbiota of mice. They found that exposure to anesthesia and surgery altered the abundance of certain bacterial species with *Escherichia*–*Shigella*, *Actinomyces*, *Ruminococcus*_*gnavus*_group, and Lachnospiraceae_FCS020_group to be enriched after anesthesia/surgery. Another study (Serbanescu et al., 2019)observed a decrease in bacterial diversity and depletion of commensal bacteria such as Clostridiales. It is notable that lower levels of Clostridiales have been associated with increased rates of infections (Becattini et al., 2017). Researchers also observed that the type of anesthetics that are used had a different impact on the changes in GM. Han et al. (Han et al., 2021)studied the effect of sevoflurane inhalation anesthesia. The intestinal microbiome of mice showed increased abundances of *Bacteroides*, *Akkermansia* and *Alloprevotella* and decreased abundancies of *Lactobacillus*.Furthermore, hypothermia during anesthesia and surgery is a relatively common occurrence in the surgical patient. Although limited research exists to link hypothermia with gut microbiota, some studies have suggested that hypothermia may contribute to changes in the composition of GM, leading to dysbiosis (Hart et al., 2011; Wang et al., 2021).

Last but not least, the operative stress can influence the composition of the microbiota and it may also be able to increase intestinal permeability through corticotropin-mediated mechanisms leading to translocation of microorganisms (Agnes et al., 2021). Generally, just the stress of surgery and injury itself, even with no use of antibiotics or other therapeutic interventions, may also decrease gut microbiota diversity (Ho et al., 2020).

## 5 Are post-operative complications related to the gut microbiota?

Postoperative complications are a serious problem, which lead to a higher morbidity and mortality rate and occur in up to 50% of patients undergoing major abdominal surgery (Trencheva et al., 2013; Young and Khadaroo, 2014; Lederer et al., 2021). Some of the most common surgical complications include surgical site infections (SSIs), anastomotic leakage (AL),postoperative ileus (POI) and malabsorption. As previously mentioned, recent studies highly suggest that surgery procedures have detrimental consequences for GM and, therefore, it is more than likely that there is an association between the patients’ gut microbiota and surgical outcomes.

### 5.1 Anastomotic leak

Anastomotic leak is a devastating problem with serious long-lasting consequences. It is defined as a defect of the intestinal wall at anastomotic site resulting in a spillage of intestinal material outside the bowel which was sutured (Rahbari et al., 2010). The cause of AL appears to be multifactorial, with surgical technique generally being the primary contributing factor. After certain surgical procedures, a leak in the connection between two structures that where surgically joined can happen. However, the exact cause is often complex and involves several factors, except for the surgery technique and type of surgery, including the characteristics of patients, such as age, sex and pre-existing medical conditions (Sciuto et al., 2018). There is compelling evidence that gut microbiota is also a risk factor for leakage (Defazio et al., 2014; Sciuto et al., 2018).

Over 60 years ago, Cohn demonstrated a direct role of GM in AL occurrence. A dog model was developed in which decontamination led to complete healing of anastomosis and AL. On the other hand, animals that received saline alone developed major leakage (Cohn and Rives, 1955). Cohen et al. (Cohen et al., 1985) in 1985, also reported a protective effect of enteric antibiotics on colonic wound healing in rats and avoidance of AL. More recently, Schardey et al. (Schardey et al., 1994) created a rat model and suggested *Pseudomonas aeruginosa* as a causative species of AL. The study was focused on esophagoduodenal AL after total gastrectomy. Olivas et al. (Olivas et al., 2012) also showed that intestinal colonization with *P. aeruginosa* led to a significant high incidence of AL in a rat model.). Hence, studies suggest that the presence of specific disruptive species may result in the development of AL.

Other studies focused on the GM synthesis in patients experiencing AL. van Praagh et al. (van Praagh et al., 2016) investigated the composition of the microbiome at the anastomosis level from patients after rectal resection. This study showed that patients with no AL had higher microbial diversity in contrast to patients who developed AL and showed less diversity with higher abundance of Lachnospiraceae.

In short, recent studied demonstrate an association between leakage and low microbial diversity, prevalence of Enterobacteriaceae and virulent microbiota (Gershuni and Friedman, 2019; Agnes et al., 2021).

### 5.2 Postoperative ileus

Postoperative ileus, a common postoperative complication, is defined as a prolonged absence of intestinal motility after surgical procedures and more often after abdominal surgery (Buchanan and Tuma, 2022).

Experimental studies and clinical observations revealed a potential link between intestinal microbiome and the pathogenesis of ileus. GM impairs intestinal peristalsis as a modulator of gut synapses or by activating dendritic cells, macrophages and monocytes. In particular, pathogenic iNOS (inducible nitric oxide synthase) produced by macrophages and monocytes, have been shown to induce POI by inhibiting smooth muscle cells. Furthermore, antibiotic administration leads to a considerable reduction of iNOS levels and, therefore, to reduced occurrence of POI, suggesting that macrophages and monocytes activation may depends on microbiota. Overall, further research is needed (Pohl et al., 2017; Bartolini et al., 2020; Agnes et al., 2021).

### 5.3 Postoperative infections

SSIs, which play a major role in postsurgical care as contributors to patient morbidity and mortality, are also highly suggested to be related to the gut microbiota. Overall, *Staphylococcus aureus* strains represent the most frequently found species in SSIs, followed by gut commensals such as *E. coli* and *E. faecalis*. In general, patients’ microbial colonization seems to be the main source of infection as microorganisms causing infectious complications are often commensals of the human body, but further research has to focus on the association between GM and SSIs development (Young and Khadaroo, 2014; Lederer et al., 2017; Bassetti et al., 2020; Lederer et al., 2021).

### 5.4 Postsurgical complications on malabsorption and overall patient nutritional level

Malabsorption refers to the impaired ability of small intestine to absorb nutrients. Major intestinal reconstructive procedures, such as Roux-en-Y gastric bypass (RYGB), ileal pouch-anal anastomosis surgery (IPAA) or pancreatoduodenectomy may contribute to postoperative malabsorption, because of the surgical operation itself and changes in GM as well. The GM plays a crucial role in absorption of nutrients and alterations in its composition, which may follow surgical procedures, lead to changes in nutrient absorption (Shi et al., 2022; Zheng et al., 2023).

Most studies reveal an increase in *Bacteroides* and Proteobacteria and a decrease in Firmicutes (Luijten et al., 2019). After laparoscopic RYGB a higher abundance of aerotolerant bacteria such as *E. coli* and *Streptococcus* are noticed and, on the other hand, after sleeve gastrectomy(SG), anaerobes, especially *Clostridium*, are more abundant (Farin et al., 2020). Sanchez-Alcoholado et al. revealed changes in the microbiota population as well, with greater levels of *Akkermansia*, *Eubacterium*, *Haemophilus*, and *Blautia* after SG and higher levels of *Veillonella*, *Slackia*, *Granucatiella*, and *Acidaminococcus* after RYGB (Sánchez-Alcoholado et al., 2019). After RYGB patients seem to be at an increased risk of malabsorption as a result of trace element deficiency and osteopenia. Furet et al. (Furet et al., 2010) suggested that high levels of Gammaproteobacteria were related to diminished nutrient absorption after RYGB. In addition, the energy-reabsorbing potential of GM tends to be decreased following laparoscopic SG, indicated by the Bacteroidetes to Firmicutes ratio (Damms-Machado et al., 2015).

Additionally, intraoperative or postoperative antibiotic administration can affect the GM and reduce the microbial diversity, leading to malabsorption (Nalluri et al., 2020; Agnes et al., 2021).

## 6 Conclusion and discussion

In the past few decades, microbiome research has increased dramatically. The development of new molecular methods such as next-generation sequencing technology helped researchers to enhance their understanding of the complicated microbiota living within the human gut. It is now known that GM changes gradually with time, as people get older, and can also be affected by multiple factors leading to great differences in the composition between individuals (Cullen et al., 2020; Tang et al., 2020). Clearly, surgical operations and, mostly, gastrointestinal surgery can profoundly affect human microbiota. This can happen as a result of disruption of the epithelial barrier during surgery and translocation of bacteria or by other perioperative practices which can alter the microbiota, such as bowel preparation and antibiotic administration (Ferrie et al., 2021). On the other hand, GM composition can affect the surgical outcome and has been described to have a crucial role in surgery complications (Agnes et al., 2021).

Consequently, surgery can alter the population of microorganisms inhabiting the GI tract and may induce an imbalance of GM. Current research examines the factors that contribute to intestinal dysbiosis and focuses on trying to find a solution. Probiotics, which are live microorganisms found in food and supplements, have been shown to improve the intestinal microbial balance and restore the GM diversity. Besides, probiotic administration seems to reduce the total length of hospital stay, the days of intensive care and, in general, the infectious and other major complications (Zhang et al., 2012; Stavrou and Kotzampassi, 2017; Mustansir Dawoodbhoy et al., 2021). Therefore, modulation of the GM with probiotics appears to be an effective method of reducing complications in patients undergoing surgery but the exact mechanisms remain unclear. Nevertheless, further studies in this field need to be done.

Overall, the results of studies indicate that GM seem to have a huge impact on surgical patients playing an important role in the development and progression of various surgical diseases. Therefore, it may be easier to predict the risk of developing of those complications and to prevent them by understanding the specific bacteria in a patient’s gut. However, more studies in larger groups of humans need to be performed for a better understanding of the role of the microbiome in surgical disease and new microbiota-based approaches to surgical care need to be examined in order to lead to new treatments and better outcomes for the patients

## Author contributions

Conceptualization, CT, SV, EB. Methodology, CT and NB. Investigation, resources AP, ZT, ES, KA. Writing—original draft preparation, AP, KA, ZT. Writing—review and editing, CT, NB, ES. Visualization, CT, SV. Supervision, EB. All authors contributed to the article and approved the submitted version.
