# Erratum: Alterations of gut microbiome following gastrointestinal surgical procedures and their potential complications

**DOI:** 10.3389/fcimb.2023.1281527

**Published:** 2023-08-31

**Authors:** Frontiers Production Office

**Affiliations:** Frontiers Media SA, Lausanne, Switzerland

**Keywords:** gut microbiota, microbiome, surgery complications, surgical disease, alterations in microbiota, peri-operative interventions

Due to a production error, two author names were incorrectly spelled as “Eugenia Stavropoulou” and “Elisavet Bezirtzoglou”. The correct spelling is “Eugenia Bezirtzoglou” and “Elisavet Stavropoulou”. The publisher apologizes for this mistake.

The original version of this article has been updated.

